# Prediction error coding as the computational basis for nocifensive and nocifensive-like behaviors

**DOI:** 10.3389/fnins.2026.1758337

**Published:** 2026-01-27

**Authors:** Alexander Batsunov, Sergei Tugin, Luisa Kirasirova, Ksenia Skobeleva, Boriss Sagalajev

**Affiliations:** Research Center for Genetics and Life Sciences, Sirius University of Science and Technology, Sirius Federal Territory, Sochi, Russia

**Keywords:** Bayesian, perception, reflex, reinforcement, salience, sensorimotor, surprise

## Abstract

Nocifensive behavior (NB) is a protective response to noxious stimuli that threaten tissue damage. However, similar motor responses, termed nocifensive-like behavior (NLB), can be evoked by unexpected innocuous stimuli. This observation challenges strict “labeled-line” models of pain, raising a fundamental question: how does the nervous system discriminate true threats from false alarms? We review evidence suggesting NB and NLB exist on a shared behavioral continuum, where defensive responses aren’t determined solely by sensory input but by the brain’s integrated threat assessment. This assessment computes the probability of harm by weighing somatosensory input against contextual factors like prior experience and multisensory cues. We propose this process is governed by a threat prediction error (TPE) mechanism, which is computationally analogous to the reward prediction error (RPE) mechanism encoded by the dopaminergic system. Under this framework, defensive responses are scaled to the magnitude of the TPE – the discrepancy between expected and actual sensory outcomes. Critically, this means the surprise of a benign touch in a dangerous environment can produce a larger TPE – and a stronger withdrawal – than the anticipation of a noxious pinprick in a safe environment. Furthermore, while NLB represents an adaptive response that can be permanently resolved as the stimulus is learned to be non-threatening, NB represents an innate response, permitting only transient suppression due to the real risk of injury. This model positions defensive behaviors as dynamic perceptual decisions arising from probabilistic inference, offering a unified theory for how context and expectation gate the expression of protective motor programs.

## Introduction

How the nervous system generates pain and protective withdrawal has been a subject of long-standing debate. The “labeled-line” theory, championed by von Frey, proposed that dedicated nociceptive neurons are hardwired to elicit pain and stereotyped nocifensive behavior (NB). This stood in contrast to “pattern” theories, which suggested pain arises from specific spatiotemporal patterns of activity across different fiber types (Dallenbach, 1939; Treede, 2016). While the discovery of specific nociceptors provided strong support for labeled lines, it became clear that this was an incomplete picture. The “gate control theory of pain” (Melzack and Wall, 1965) was a pivotal pattern theory that integrated inhibition from non-nociceptive fibers, highlighting the importance of integration in the spinal cord. Modern models synthesize these views, recognizing a labeled-line input from nociceptors, but with central processing that is subject to profound gain control and modulation by context (Treede, 2016).

This modern synthesis is challenged, however, by the common observation: similar, often indistinguishable, motor responses can be triggered by unexpected but entirely innocuous stimuli. A tap on the shoulder in a quiet room, the sensation of an insect crawling on the skin, or the classic example of tickle can all evoke a startle, a jump, or a swift withdrawal. We term this phenomenon nocifensive-like behavior (NLB), posing a fundamental question for existing pain models: how does the nervous system dynamically discriminate true threats from false alarms? If the same motor program can be engaged by both noxious and innocuous inputs, the brain must be performing an integrated assessment that goes beyond simple sensory input. We argue that defensive behaviors are not merely reflexive but are dynamic perceptual decisions (Borsook et al., 2013; Edwards et al., 2012). They arise from a probabilistic inference process that weighs incoming somatosensory data against a rich context of prior experience, expectation, and multisensory cues (Büchel et al., 2014).

In this theoretical work, we unify diverse evidence to propose that NB and NLB exist on a shared behavioral continuum, governed by a computational mechanism analogous to reward prediction error (RPE) coding (Dabney et al., 2020). We introduce the concept of a threat prediction error (TPE), where the magnitude of a defensive response is scaled to the discrepancy between the expected and actual sensory outcome. A key prediction is that a person who is startled by an unexpected tap in a dark alley may exhibit a more forceful defensive reaction (NLB) than a participant in a controlled experiment who anticipates and consents to a calibrated pinprick (NB). The former scenario maximizes the TPE by violating a strong threat prior with a benign input, while the latter minimizes it by aligning a noxious input with a weak or absent threat prior. We first review the evidence for the NB/NLB continuum and the role of context. We then explore how dopaminergic (DA) circuitry, known for encoding RPE, provides a plausible neural substrate for TPE computation. Finally, we present our TPE model as a unified theory for how the brain gates the expression of protective motor programs, framing them as the output of a continuous probabilistic assessment of risk.

## Defining the behavioral continuum

Noxious stimulation represents the extreme end of a somatosensory continuum – the highest intensity that primary afferents can encode. When applied to glabrous skin, noxious mechanical stimulation activates nociceptive afferents (high-threshold mechanoreceptors), thereby evoking NB. Meanwhile, concurrent activation of non-nociceptive afferents (low-threshold mechanoreceptors, LTMRs) coordinates NB execution (Arcourt et al., 2017). Notably, even in its simplest form (e.g., the flexion reflex), NB is not hardwired to produce a stereotyped withdrawal at a fixed latency. Instead, it represents a dynamically modulated behavior shaped by competing motor demands: rapid hindlimb withdrawal vs. postural stability (Browne et al., 2017; Callahan et al., 2008). Furthermore, brainstem motor responses (e.g., vibrissa movement) can precede spinal motor responses, suggesting that NB prioritizes danger localization to guide withdrawal direction (Browne et al., 2017). Finally, the unpredictability of either the timing or intensity of a noxious stimulus evokes heightened withdrawal responses, but their combined unpredictability produces an even greater response amplitude (Jure et al., 2020). Descending pronociception aligns with the principle that NB is prioritized when threats are sudden; conversely, when the brain anticipates danger (as in a fight-or-flight state), NB may engage descending antinociception to favor survival-enhancing strategies, even if those strategies carry inherent risks (Donaldson and Lumb, 2017).

In contrast, innocuous stimulation engages only non-nociceptive afferents, yet their activation is sufficient to trigger NLB in fully acclimated mice (Abdus-Saboor et al., 2019). While mechanically evoked NLB in the form of hindlimb withdrawal occurs with longer latency (~100 ms) than true NB (~50 ms), it can still be accompanied or even preceded by a head turn. Moreover, pain-associated responses (orbital tightening, jumping, paw shake, and paw guarding) are not exclusive to NB but rather exhibit a significantly higher incidence than during NLB (Abdus-Saboor et al., 2019). Notably, spinally projecting neurons in the rostral ventromedial medulla (RVM) – classically characterized as pronociceptive ON and antinociceptive OFF cells in anesthetized preparations – exhibit a broader dynamic range in awake animals. While they still respond to noxious stimuli, they also react to innocuous stimuli, provided the latter are unexpected (Foo and Mason, 2003). Interestingly, RVM neurons react not only to somatosensory but also to auditory (Leung and Mason, 1999; Oliveras et al., 1990, 1989) and visual stimuli (Leung and Mason, 1999), suggesting NLB arises from cross-modal integration.

These findings indicate that NB and NLB exist along a shared behavioral continuum. Rather than being strictly determined by the activation of nociceptive vs. non-nociceptive afferents, the initiation of defensive motor responses depends more critically on the brain’s integrated threat assessment. This assessment weighs somatosensory input against contextual factors – including prior experience and multisensory cues – to compute the probability of harm. Crucially, the distinction between NB and NLB lies in their persistence: while NLB is adaptive, diminishing as the brain learns the stimulus is innocuous, NB is innate and cannot be permanently suppressed due to the high risk of sustaining injury.

Critically, somatosensory stimuli often co-activate multiple afferent types, and their interactions, rather than independent labeled-line signaling, shape perceptual outcomes (Prescott et al., 2014). For instance, unnatural innocuous stimuli (e.g., the thermal grill illusion) can disinhibit nociceptive pathways, leading to pain and associated NB despite the absence of true tissue damage (Craig and Bushnell, 1994). Conversely, even when nociceptive pathways remain inhibited, overly synchronous activation of LTMRs may evoke paresthesia and associated NLB (Sagalajev et al., 2024). This is evident during electrical stimulation of the peripheral nerve below motor threshold, where the resulting NLB constitutes a behavioral response to the perceived paresthesia (unnatural, tingling sensation) rather than resulting from direct motoneuron activation or representing the Hoffman reflex (Polus et al., 1991). Notably, while NB evoked by the thermal grill resists adaptation due to persistent nociceptive pathway engagement, electrically evoked NLB may subside if the brain learns paresthesia is non-threatening (Mortaza et al., 2023).

Another compelling example is tickle, an innocuous stimulus that robustly evokes NLB despite activating only LTMRs under natural conditions (i.e., without excessive synchrony as during paresthesia or nociceptive pathway disinhibition as during the thermal grill illusion). Strikingly, self-tickling not only fails to elicit NLB but can even suppress it when caused by an external tickle (Proelss et al., 2022). This dissociation highlights two critical determinants of NLB generation: the dynamic quality of the stimulus (e.g., unpredictability in timing, location, and intensity) and the absence of congruent proprioceptive feedback, which likely enables the brain to distinguish self-generated from external touch. These observations align with recordings in awake rodents, where RVM neurons respond to unexpected external touch but remain unmodulated during self-grooming (Foo and Mason, 2003). Unlike other forms of NLB, which diminish as the brain classifies the stimulus as innocuous, tickle-evoked NLB persists for the duration of stimulation, suggesting that the brain continuously processes its evolving sensory features without fully habituating.

Notably, while NB is usually associated with screaming, NLB, as seen with tickle, can also be associated with laughter. Though the neural mechanisms of laughter remain elusive, its emergence may align with the “incongruity detection and resolution” theory of humor, wherein laughter arises from moderate unpredictability, such as a joke’s punchline being neither fully predictable nor entirely nonsensical (Vrticka et al., 2013). Similarly, tickle may represent a “somatosensory joke,” where the brain interprets dynamic, innocuous stimuli as playful rather than threatening. However, this effect is critically dependent on the social context. If the tickle is perceived as originating from a non-familiar or threatening source, laughter may give way to screaming (Fridlund and Loftis, 1990), mirroring other NLB scenarios where perceived threat overrides the benign nature of the input.

The dynamic interplay between NB and NLB suggests that defensive responses are not merely reflexive but are governed by predictive processes that compare sensory input with internal models of threat. This raises the possibility that such behaviors are modulated by prediction errors – discrepancies between expected and actual sensory outcomes – akin to those encoded by the DA system in reward learning. Just as reward prediction errors (RPEs) drive appetitive behavior, threat prediction errors (TPEs) may calibrate defensive responses, scaling their magnitude to the degree of unexpected harm. In the following section, we explore how DA-dependent circuits, particularly those involved in reinforcement learning, may provide a computational framework for understanding how NB and NLB are gated by probabilistic threat assessment.

## Temporal dynamics in a shared circuit for NB and NLB

The phenomenological similarity between NB and NLB suggests they are mediated by a shared neural circuitry. This view is consistent with modern frameworks that have moved beyond the historical labeled-line vs. pattern theory debate. Contemporary models view the nociceptive system as a complex hierarchy of gain control mechanisms, where spinal and supraspinal circuits integrate ascending signals with descending modulatory influences to shape the final perceptual and behavioral output (Treede, 2016). Specifically, we propose that the distinction between NB and NLB arises not from separate, hardwired circuits, but from the differential engagement of a common defensive network – including components traditionally considered nociceptive – in response to both noxious and innocuous stimuli.

It is important to note, however, that a significant challenge in interpreting the neural circuitry of pain and defense is that much of our foundational knowledge comes from studies conducted in anesthetized or physically restricted animals. While these approaches are necessary for stable electrophysiology and imaging, they preclude the observation of naturalistic behavior and the brain’s full repertoire of state-dependent neural responses. Thus, a critical synthesis of existing data is required to bridge this gap and build a model for ethologically relevant circuit function.

**Figure 1:**
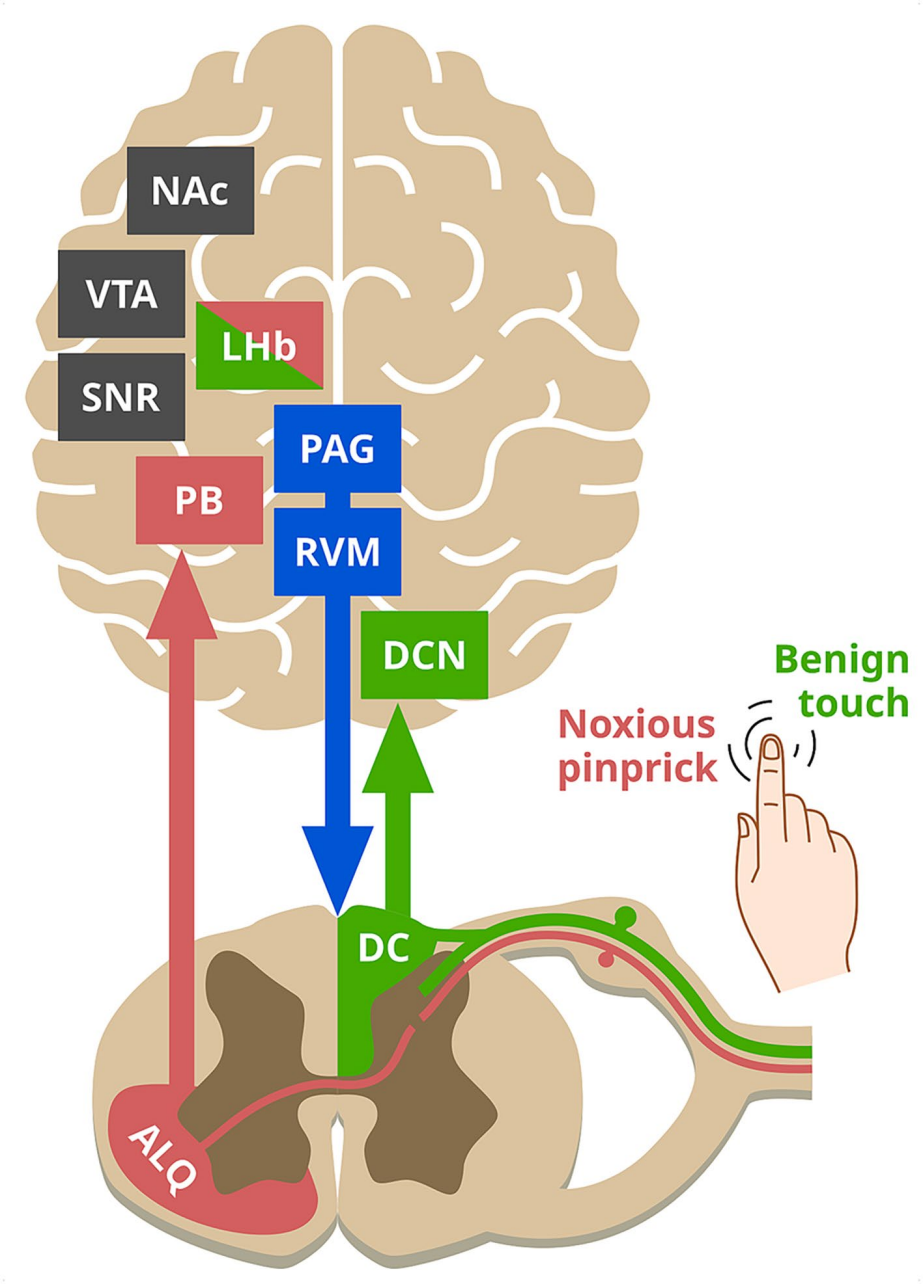
A schematic of the proposed core circuit for computing and implementing the TPE. Colors: black, dopaminergic circuitry; blue, descending modulation of spinal circuitry; green, non-nociceptive circuitry; red, nociceptive circuitry. Neural structures: ALQ, anterolateral quadrant; DC, dorsal column; DCN, dorsal column nuclei; LHb, lateral habenula; NAc, nucleus accumbens; PAG, periaqueductal gray; PB, parabrachial nucleus; RVM, rostral ventromedial medulla; SNR, substantia nigra reticulata; VTA, ventral tegmental area.

The parabrachial nucleus (PB) serves as a relay for transmitting nociceptive signals from the anterolateral quadrant of the spinal cord to midbrain DA regions (Figure 1), including the substantia nigra reticulata (SNR) and ventral tegmental area (VTA). Noxious mechanical and thermal stimulation of glabrous skin activates parallel PB projections to both the SNR and VTA. Since SNR subsequently inhibits DA release from VTA projections to the nucleus accumbens (NAc), this results in reduced reward-seeking behavior in mice (Yang et al., 2021). Intriguingly, PB → SNR projections also exhibit weak responses to innocuous mechanical and thermal stimuli in awake, head-fixed mice (Yang et al., 2021). This raises the possibility that the PB may relay both noxious and innocuous tactile signals to mesolimbic DA regions in unrestrained animals. A likely source for the putative innocuous input to the PB is the dorsal column (DC) pathway of the spinal cord. While a direct connection has not been established, functional studies demonstrate that DC activation suppresses cocaine-induced c-fos expression in the NAc via a polysynaptic circuit (Chang et al., 2017). This DC-mesolimbic pathway engages the lateral habenula, a hub for both noxious and innocuous stimuli with direct projections to the VTA.

Further evidence for DA involvement in innocuous stimulus processing comes from primate studies. In awake monkeys, innocuous vibrotactile stimulation of the fingertip enhances midbrain DA neuron firing, but only when the stimulus is consciously detected (de Lafuente and Romo, 2011). Strikingly, while the primary somatosensory cortex encodes tactile input within ~30 ms, DA neurons respond later (~150 ms), suggesting their activity reflects perceptual outcomes (de Lafuente and Romo, 2012). Furthermore, studies of classical conditioning demonstrate that midbrain DA neurons in monkeys are activated by visual cues, irrespective of whether they predict appetitive (food), neutral (none) or aversive (air puff) outcomes. The critical distinction lies in the response magnitude: while responses to appetitive cues are the strongest, the phasic elevations in discharge rate to neutral and aversive cues are evident and are remarkably similar in magnitude (Joshua et al., 2008). Crucially, when the actual outcomes are delivered, the DA response evolves through a temporal sequence. An initial, short-latency activation (40–120 ms) reflects raw sensory intensity, independent of stimulus valence. It is only in the subsequent phase (150–250 ms) that the influence of subjective value becomes dominant, with sustained activation for rewards and suppression for aversive and neutral events (Fiorillo et al., 2013).

Together, these findings demonstrate that midbrain DA neurons function as a unified salience detector, activated by behaviorally relevant events across the motivational spectrum. The critical insight is that the initial DA response represents a non-specific “alert” signal, while subsequent DA activity encodes value. We propose that this initial alert signal carries the unsigned salience necessary for a TPE, which is then refined by downstream circuits to guide the appropriate defensive response.

Importantly, direct evidence for midbrain DA coding of a TPE-like signal comes from a recent study (Cheng et al., 2025), showing that midbrain DA neurons dynamically change their response to aversive stimuli, shifting from signaling initial novelty response to relief, and finally to the predictive onset of the event to guide escape via the periaqueductal gray (PAG). This finding aligns with and complements the well-established role of the PAG in fear conditioning, where it not only generates fear behavior but can also estimate threat probability (Wright and McDannald, 2019). Resembling the TPE-like properties of midbrain DA neurons, the PAG exhibits elevated firing to unexpected vs expected punishment and habituates as shocks become predictable (Johansen et al., 2010; McNally et al., 2011). According to the prevailing model, these TPE signals are generated in the PAG when value expectations – encoded by a network of forebrain regions – are compared with primary nociceptive inputs (Roy et al., 2014). Given their reciprocal connections (Breton et al., 2019; Ntamati et al., 2018), the midbrain DA regions and PAG are strong candidates for the proposed core circuit that computes TPE. This TPE signal can then be directly relayed from the PAG to the RVM (van Bockstaele et al., 1991) to calibrate the expression of NB and NLB.

## Gating defense with prediction errors

DA signaling serves as a computational basis for the temporal difference (TD) theory of reinforcement learning. The TD theory describes how the brain predicts future outcomes by continuously comparing actual sensory input with prior expectations, updating its models through RPEs. While the classical TD theory operates on a single quantity, representing the average over all possible reward outcomes, recent advances in computational neuroscience reveal a more sophisticated variant – the distributional TD theory – where midbrain DA neurons encode a complete probability distribution of possible outcomes (Dabney et al., 2020). This allows the brain to simultaneously represent optimistic, pessimistic, and neutral expectations, forming a multidimensional predictive framework that guides motivated behavior.

**Figure 2:**
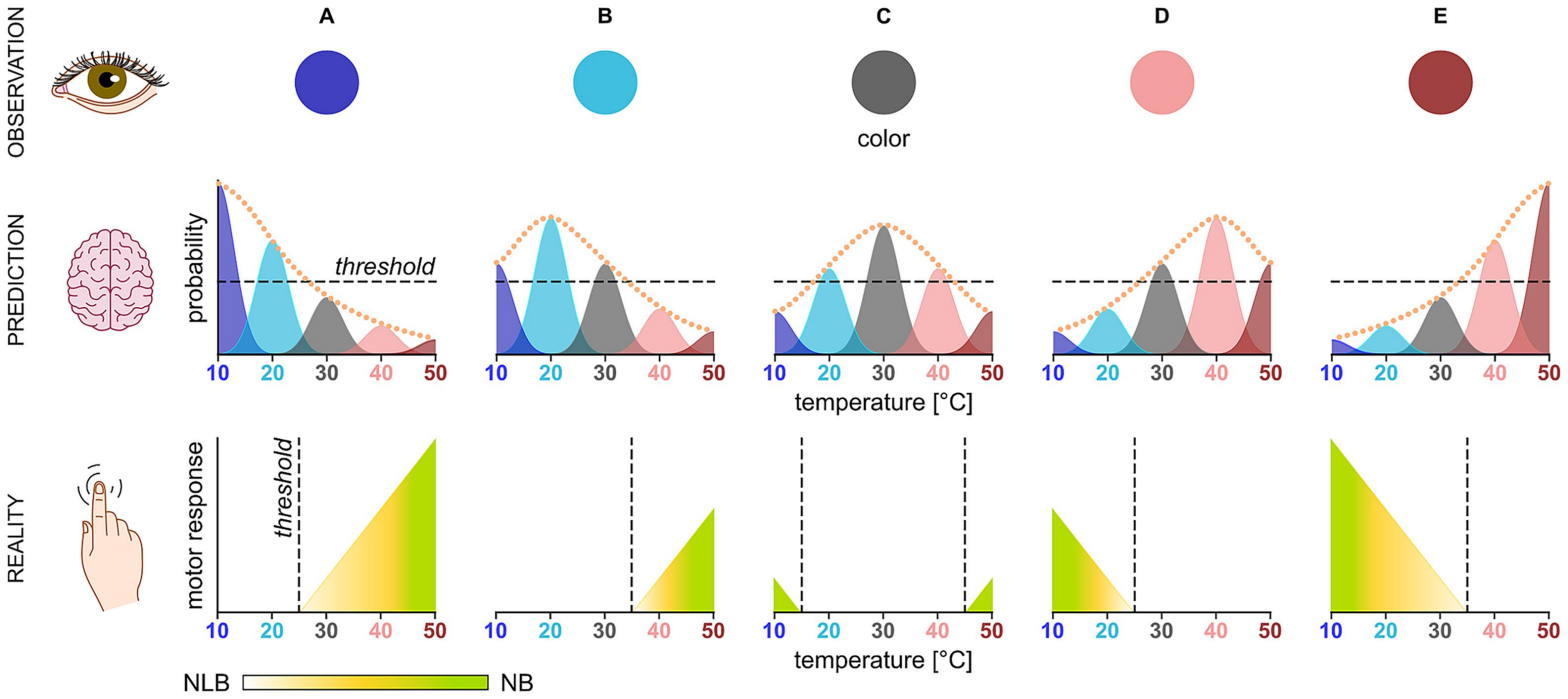
Predictive mismatch between visual context and somatosensory feedback scales defensive behavior. Visual features in the top row (observation) generate prior probabilities for expected surface temperature in the middle row (prediction), which are compared to somatosensory feedback in the bottom row (reality). In each scenario **(A–E)**, the brain distributes probabilities over a temperature range, peaking at the subjectively most likely temperature for the given color. Accurate predictions (above the horizontal dashed line) suppress motor responses, while mismatches (below the horizontal dashed line) generate a TPE that scales the response magnitude. This response starts at the vertical dashed line and ranges from NLB (yellow) to NB (green), depending on whether the sensed temperature is innocuous (between 15 °C and 43 °C) or not. For graphical clarity, the prior is represented by its probability at five discrete temperatures, though the underlying model is a continuous, unimodal probability density function (orange dotted line).

Extending the distributional TD theory of reinforcement learning, we propose that NB and NLB are governed by a computationally analogous system, potentially mediated by the same DA-dependent neural circuitry. Just as the DA system encodes reward distributions, an analogous system could compute probabilities that a stimulus possesses a range of threat values based on multimodal cues (Figures 2A–E). For instance, visual input – such as a blue hue of a steel stove – could generate a prior distribution assigning the highest probability to a thermally cool surface, moderate probability to adjacent temperatures, and the lowest probability to all other temperatures, based on learned associations (Figure 2B). Meanwhile, somatosensory feedback would provide direct evidence. If the vision-based prediction matches the somatosensory input (the surface feels as cool as it appears), withdrawal is suppressed. However, a mismatch between predicted and actual values would generate a TPE, triggering a withdrawal proportional to its magnitude. Critically, even if one modality (e.g., temperature) is predicted accurately, concurrent mismatches in other modalities (e.g., texture) could still elicit the withdrawal, reflecting a system that integrates distributed threat expectations across multiple sensory channels to gate defensive actions.

The above hypothetical example works because the visual cue (blue hue) gave assurance that the stove is more likely cool than warm. If, however, the color were gray, the peak of the probability distribution would shift toward a more neutral temperature (Figure 2C). In this case, tactile information indicating that the stove is moderately cool or warm would be less likely to trigger withdrawal, as the mismatch would remain marginal. A gray surface would likely elicit withdrawal only if the mismatch were substantial – extending into the nociceptive temperature range (below 15 °C or above 43 °C).

This reliance on visual cues to shape tactile expectations aligns with findings in rodents that the brain actively combines visual and tactile signals to form a more accurate percept than either modality could provide alone (Nikbakht et al., 2018). While convergence of sensory channels enables richer perceptual experiences, it also creates inherent ambiguities in neural coding (Diamond, 2019). A single physical feature of the stimulus often contributes to multiple perceptual dimensions, leading to systematic confounds. For example, in rats, the same vibratory signal cannot be parsed into separate amplitude and frequency components; instead, animals perceive their product, creating a fundamental ambiguity where neither feature can be judged independently (Adibi et al., 2012). Similarly, in our model, the violation of a cross-modal experience (visually cool but tactilely warm or visually neutral but tactilely hot) generates a significantly large TPE to drive NLB and NB, respectively.

Another scenario to consider is when the stove is red (Figure 2E), and a person deliberately suppresses withdrawal responses to check its temperature. Even with the engagement of descending antinociception, NLB may still occur upon touching if the stove is unexpectedly tepid or cool. This principle finds support in EEG recordings of healthy subjects, where an unexpected innocuous stimulus presented after a series of noxious stimuli elicits a mismatch negativity component, a canonical signature of prediction error. It can appear as early as 64 ms post-stimulus, demonstrating a rapid, pre-conscious evaluation (Zhao et al., 2015). Furthermore, descending antinociception is often modality-specific (Bourbia et al., 2014). Hence, the voluntary suppression of NB to noxious heat may not generalize to noxious cold (Apte et al., 2025).

The notion that visual cues generate priors is demonstrated empirically by studies where colors of high hue-temperature (e.g., red) increase the perceived intensity of cool innocuous stimuli, while colors of low hue-temperature (e.g., green) decrease the perceived intensity of innocuous warmth and noxious heat (Landgrebe et al., 2008). Aside from changing the sensory experience quantitatively (by altering perceived intensity), these priors can transform it qualitatively. For instance, a noxious cold stimulus paired with a red cue is perceived as more painful and as having a more burning quality than when paired with a blue one (Moseley and Arntz, 2007). Furthermore, such cross-modal effects are contingent on the visual context being integrated into the body’s self-representation, as demonstrated by thermal illusions elicited through the rubber hand paradigm (Durgin et al., 2007; Kanaya et al., 2012).

## Discussion

In this theoretical proposition, we have integrated existing evidence to argue that NB and NLB are not the products of separate, labeled-line circuits but are distributed along a continuum governed by a unified computational principle: TPE. This framework repositions the brain from a passive sensory relay into an active inference system, where a failure to accurately predict sensory events is interpreted a priori as a potential threat, triggering a proportional defensive response. The TPE model offers a parsimonious explanation for a wide range of phenomena, from the vigor of a withdrawal from an unexpected tickle to the suppression of pain responses in high-stakes survival situations.

The primary strength of the TPE model is its ability to unify disparate observations under a single computational umbrella. It explains why the same motor program can be engaged by both noxious and innocuous stimuli – the key variable is not the stimulus quality per se, but the magnitude of the discrepancy between the expected and actual sensory events. Crucially, the expected event is not a simple scalar but a rich, context-defined prior distribution of all possible scenarios. Our framework, grounded in distributional TD theory (Dabney et al., 2020), posits that multidimensional threat appraisals – including controllability, imminence, escape potential, and safety signaling – determine the shape (mean, variance, skew) of this prior distribution. The TPE is therefore computed as the mismatch between this multidimensional prior and the incoming feedback from sensory channels. This reconciles the seemingly paradoxical finding that a high-context NLB (e.g., a surprise touch in a dangerous environment) can be more vigorous than a low-context NB (e.g., an expected pinprick in a safe lab setting). By leveraging the well-established formalisms of reinforcement learning, the TPE model provides a quantitative framework for future experiments, moving the field beyond purely descriptive accounts.

The TPE can be understood as the instantiation of the sensory surprise signal described by Press et al. (2020) in terms of the Kullback–Leibler Divergence (KLD). In their Opposing Process theory, perception is optimized through two successive stages. First, a Bayesian process biases perception toward expected stimuli to resolve sensory ambiguity, analogous to our example where a blue hue biases temperature perception toward coolness. Second, when sensory evidence strongly contradicts predictions (high KLD), catecholaminergic release upweights the processing of unexpected signals to facilitate learning. This second stage corresponds to the generation of a large TPE when a blue object feels unexpectedly warm. Our contribution extends the Opposing Process theory by specifying its neural implementation and behavioral consequences. We propose that midbrain DA neurons, through distributional TD coding, compute this KLD (i.e., TPE) signal. Crucially, a sufficiently large KLD (i.e., TPE) signal not only enhances perceptual processing as described by Press et al., but also gates defensive motor outputs (NB/NLB). This implements a rapid “better-safe-than-sorry” strategy (Van den Bergh et al., 2021), where large sensory surprises are treated as potential threats by default. The motor program thus engaged serves dual adaptive functions. Pragmatically, it minimizes immediate harm by removing the body from potential danger. Epistemically, it constitutes an active inference maneuver: by altering sensory input (i.e., stopping the tactile sensation) and enabling new sensory sampling (e.g., more vigilant visual inspection), it helps resolve the uncertainty that triggered the TPE.

Although the examples provided thus far emphasize discrepancies involving visual and tactile inputs, the TPE framework is well-suited to compute mismatches across any integrated sensory channels. Defensive gripping on a rollercoaster (visual-vestibular mismatch), nausea from a revolting sight (visual-visceral mismatch), or a startle in response to an incongruent self-reflection in a mirror (visual-proprioceptive mismatch) all illustrate this principle. Critically, the same computational logic applies to non-visual contexts as well, such as the disruption of rhythmic typing when a keypress is met with unexpected silence. These examples underscore that TPE-driven defensive responses are triggered by the violation of multimodal expectations, regardless of which sensory channel carries the surprise. Moreover, while we have emphasized somatic motor responses for clarity, the full expression of a defensive behavior inherently involves coordinated engagement of the autonomic nervous system. These autonomic responses are a critical component of resolving the surprising event, modulating sensory precision and preparing the body for action. In contexts where overt movement is constrained, autonomic responses may in fact represent the primary output of the TPE system.

A key deduction from the TPE framework is that NLB is adaptive while NB is innate, which warrants further explanation. Both NLB and NB can be facilitated, as seen in chronic pain patients with allodynia and hyperalgesia, respectively. However, in healthy subjects, only NLB can be fully resolved permanently. We propose that this distinction stems from the nociceptive system’s unique organization. Unlike sensory systems that rely on hierarchical, serial processing to extract complex features (e.g., discriminative touch), the nociceptive system operates through a highly distributed, parallel architecture (Coghill, 2020). Multiple neural structures can encode noxious stimulus intensity independently, creating a robust and redundant network for pain signaling. This is evident in clinical observations: while lesions to the primary somatosensory cortex abolish fine tactile discrimination, pain perception persists. Furthermore, pain often remains intact even after more large-scale brain lesions, including lobotomy and hemispherectomy (Coghill, 2020). Consequently, in healthy subjects, NB can be suppressed only temporarily (e.g., for immediate survival), as driving its TPE to zero inevitably leads to a rebound. In relationship to DA firing, this distinction is supported by research in monkeys showing that responses of midbrain DA neurons to innocuous stimuli are context-dependent, while responses to noxious stimuli are unconditional (Romo and Schultz, 1989).

A profound implication of the TPE model is that the core symptoms of chronic pain – allodynia and hyperalgesia, as well as their associated behaviors (NLB and NB, respectively) – can be reconceptualized as disorders of miscalibrated prediction (Castejón et al., 2024). In this view, a pathologically biased prior generates expectations that are easily violated, leading to excessively large TPEs from sensory inputs that are objectively benign or only moderately noxious. This results in a sustained state of negative affective charge, a formal computational signal representing diminished confidence in one’s own predictive model (Hesp et al., 2021). Consequently, the system exists in a persistently anxious state, which may explain the high comorbidity between chronic pain and anxiety disorders (Traxler et al., 2022). Clinically, this implies that rather than simply blocking nociceptive signals, treatments could aim to recalibrate the brain’s predictive model. Techniques like graded exposure therapy (López-de-Uralde-Villanueva et al., 2016), perceptual retraining (Foell et al., 2014; Ashar et al., 2022), and virtual reality (Alemanno et al., 2019) could be seen as methods to systematically generate small, manageable TPEs in a safe context. Furthermore, our model suggests that therapies targeting the DA system or its connected circuits, potentially via neuromodulation (Kim et al., 2008) or neurofeedback (Orakpo et al., 2021), can help normalize aberrant TPE signaling in chronic pain.

In summary, by building on modern models that have moved beyond strict labeled-line concepts, we see that the vigor of a defensive reaction is a measure of the brain’s surprise, not just the stimulus’s intensity. The TPE framework integrates different dimensions of threat appraisal to shape the prior distribution of expected sensory inputs, which, if violated, produces a risk-averse behavior, comprising both motor and autonomic components. This not only unifies a wide range of defensive repertoires but also redefines them as products of dynamic sensory inference, opening the door to a deeper, computationally grounded understanding of how we navigate a world of potential harm.

### Limitations

While the TPE framework provides a coherent unifying model for defensive behaviors, several limitations must be acknowledged.

First, our model is primarily a theoretical synthesis. While it integrates evidence across species and methodologies, its core computational and circuit-level mechanisms require direct empirical validation. Future experiments must test specific predictions, such as recording from midbrain DA or PAG neurons during paradigms that manipulate threat expectation and measure NLB/NB, to confirm they encode a TPE signal that scales with defensive response vigor.

Second, the model heavily emphasizes the role of DA midbrain circuits and their connections to the PAG and RVM. While this pathway is strongly supported by existing data, defensive behaviors are governed by a vastly distributed network. Our focus may underrepresent the contributions of other critical regions – such as the amygdala, insula, anterior cingulate cortex, and cerebellum – in forming threat expectations, assessing context, and modulating spinal reflexes. The TPE framework logically implies that such regions are sources of prior information, but their specific computational roles and connections to the midbrain-PAG-RVM axis remain to be defined.

Third, our framework borrows formalisms from reinforcement learning, particularly the distributional TD theory, which was developed to explain reward-based learning. While the computational parallels are compelling, the direct applicability of these algorithms to the threat domain is not yet proven. Key differences may exist, such as the timescales of prediction (imminent threat vs. delayed reward) or the neural implementation (potentially distinct cell populations or neuromodulators). The model assumes a degree of symmetry between reward and threat processing that may not hold in detail.

## Data Availability

The original contributions presented in the study are included in the article/supplementary material, further inquiries can be directed to the corresponding author.
